# 

**Published:** 1863-07

**Authors:** 


### CONTENTS.

### ORIGINAL CONTRIBUTIONS.

ART. XV. Report on Typhoid Fever. By H. Noble, M.D., of Heyworth, Ill., ........ 289
ART. XVI. Report of the Special Committee on Diseases of the Eye. By E. L. Holmes, M.D., of Chicago. One of the Surgeons of the Chicago Charitable Eye and Ear Infirmary, ........ 296
ART. XVII. Cerebro-Spinal Meningitis. By N. S. Davis, M.D., Professor of Clinical Medicine, &c., ........ 304
ART. XVIII. Dislocation of Ankle, with Compound Fracture of the Tibia and Fibula. Reported by James S. King, M.D., of Lemont, Illinois. Formerly Resident Physician St. John's Hospital, Cincinnati, Ohio, ........ 315

### SELECTIONS.

Lectures on Diseases of the Eye. By Henry D. Noyes, M.D., Assistant-Surgeon New York Eye Infirmary, ........ 318
Lectures on New Remedies and their Therapeutical Applications. By Samuel R. Percy, M.D., Prof. of Materia Medica and Therapeutics, ........ 327
An Organic Base without Oxygen, ........ 314
Pus in the Blood; No Metastatic Abscesses, ........ 333

### BOOK NOTICES

A Practical Handbook of Medical Chemistry. By John E. Bowman, F.C.S., formerly Professor of Practical Chemistry in King's College, London. Edited by Chas. L. Bloxam, Professor of Practical Chemistry in King's College, London, ........ 334
The Medical Student's Vade-Mecum. By George Mendenhall, M.D., Professor of Obstetrics and Diseases of Women and Children in the Medical College of Ohio, &c., ........ 334
A Practical Treatise on Fractures and Dislocations. By Frank Hastings Hamilton, A.B., A.M., M.D., &c., ........ 334
The Action of Medicines in the System. By Frederick William Headland, M.D., B.A., F.L.S., Licentiate of the Royal College of Physicians, &c., &c., ........ 334
A Theoretical and Practical Treatise on Midwifery, including the Diseases of Pregnancy and Parturition, and the Attentions required by the Child from Birth to the Period of Weaning. By P. Cazeaux, Member of the Imperial Academy of Medicine, &c., Paris. Translated by Wm. R. Bullock, M.D., ........ 334

### EDITORIAL.

Mercy Hospital, ........ 335
Medical Department of Lind University, ........ 336
Report on the Sanitary Condition of the West Division of the City. By H. Wanzer, M.D., Chicago, ........ 340
Calomel and Tartar Emetic as Remedial Agents, ........ 342
Statistics of the Chicago Eye and Ear Infirmary, ........ 345
The Pitcher Plant in Small Pox, ........ 346
Chronic Eczema, ........ 347
On Gonorrhœal Ophthalmia, ........ 348
Syphilis, ........ 348
Insane Poor,—Colocynth, ........ 349
Breast-pin Swallowed by a Child, ........ 349
Former and Present Mortality of London, ........ 349
Oxygen Gas, ........ 350
Extraction of a Ball, ........ 350
Poisonous Ballet Dresses, ........ 350
Elaterium,—Muscular Electricity, ........ 350

